# An integrative approach to ortholog prediction for disease-focused and other functional studies

**DOI:** 10.1186/1471-2105-12-357

**Published:** 2011-08-31

**Authors:** Yanhui Hu, Ian Flockhart, Arunachalam Vinayagam, Clemens Bergwitz, Bonnie Berger, Norbert Perrimon, Stephanie E Mohr

**Affiliations:** 1 Drosophila RNAi Screening Center, Department of Genetics, Harvard Medical School, 77 Avenue Louis Pasteur, Boston, MA 02115, USA; 2 Endocrine Unit, Massachusetts General Hospital, 50 Blossom Street, Boston MA 02114, USA; 3 Math Department and Computer Science and Artificial Intelligence Laboratory, MIT, 77 Massachusetts Avenue, Cambridge, MA 02139, USA; 4 Howard Hughes Medical Institute, 77 Avenue Louis Pasteur, Boston, MA 02115, USA

## Abstract

**Background:**

Mapping of orthologous genes among species serves an important role in functional genomics by allowing researchers to develop hypotheses about gene function in one species based on what is known about the functions of orthologs in other species. Several tools for predicting orthologous gene relationships are available. However, these tools can give different results and identification of predicted orthologs is not always straightforward.

**Results:**

We report a simple but effective tool, the *Drosophila *RNAi Screening Center Integrative Ortholog Prediction Tool (DIOPT; http://www.flyrnai.org/diopt), for rapid identification of orthologs. DIOPT integrates existing approaches, facilitating rapid identification of orthologs among human, mouse, zebrafish, *C. elegans, Drosophila*, and *S. cerevisiae*. As compared to individual tools, DIOPT shows increased sensitivity with only a modest decrease in specificity. Moreover, the flexibility built into the DIOPT graphical user interface allows researchers with different goals to appropriately 'cast a wide net' or limit results to highest confidence predictions. DIOPT also displays protein and domain alignments, including percent amino acid identity, for predicted ortholog pairs. This helps users identify the most appropriate matches among multiple possible orthologs. To facilitate using model organisms for functional analysis of human disease-associated genes, we used DIOPT to predict high-confidence orthologs of disease genes in Online Mendelian Inheritance in Man (OMIM) and genes in genome-wide association study (GWAS) data sets. The results are accessible through the DIOPT diseases and traits query tool (DIOPT-DIST; http://www.flyrnai.org/diopt-dist).

**Conclusions:**

DIOPT and DIOPT-DIST are useful resources for researchers working with model organisms, especially those who are interested in exploiting model organisms such as *Drosophila *to study the functions of human disease genes.

## Background

It is well established that the evolutionary conservation of proteins correlates with conservation at the level of biological and/or biochemical functions. Thus, identification of putative orthologs among species can be a helpful first step in functional genomics studies, as functional information obtained in one species can be used to formulate testable hypotheses about function in another. Indeed, for many approaches, identification of orthologs is the first step to using model systems such as *S. cerevisiae*, *C. elegans*, and *Drosophila melanogaster (Drosophila) *to study human genes, such as the set of disease genes in the Online Mendelian Inheritance in Man (OMIM) database [1-4] or the results of a genome-wide association study (GWAS) [5,6]. Similarly, orthologs of genes functionally characterized in model organisms may identify as yet unknown candidate genes relevant to human health. Thus, in addition to its value in evolutionary studies, mapping of orthologs among species can serve important purposes for functional analyses.

To date, a large number of approaches and corresponding databases have been developed to predict putative orthologous genes among various species. Most of these are based on either phylogeny-based algorithms (*e.g*. TreeFam, Phylome, Ensembl Compara) or on sequence similarity (*e.g*. InParanoid, orthoMCL and OMA) [7-12]. More recently, additional tools have been reported based on functional similarity among ortholog groups identified using protein-protein interaction (PPI) networks (*e.g*. NetworkBLAST, IsoBase) [13-15]. Notably, PPI-based tools have strong potential to improve in performance over time, as PPI data sets are improving in coverage and quality at a rapid pace. Because of their differing approaches, user interfaces, output formats and so on, these otherwise powerful tools and algorithms can be difficult to use by a biologist without expertise in bioinformatics, particularly when handling large-scale data sets such as the results of a full-genome RNAi screen or large-scale proteomics analysis.

Furthermore, determining the sensitivity and specificity of a given ortholog prediction approach is not easy. Only a few studies have compared the relative quality of results obtained with different tools. Evaluation efforts are hindered by the very reason that motivates a researcher to develop an ortholog prediction tool--*i.e*. we simply do not have enough functional data. Nevertheless, some studies have looked at the comparative quality of some tools using available approaches. Altenhoff *et al*., for example, assessed several tools, including OMA, orthoMCL, Inparanoid and Compara, using gene ontology (GO), enzyme category (EC), gene expression profile and gene neighborhood conservation. The results of their comparison and corresponding estimate of the relative quality of the tools suggest that simple bidirectional best hit (BBH)-based ortholog predictions are more likely to yield functionally consistent predicted orthologs. Their results also highlight that different tools have different advantages [16].

As each of the existing tools has different strengths, combining results generated by more than one tool might be the best way forward. At least two such integrative methods have been reported. HCOP is a human-centered ortholog prediction tool that in its original published form, facilitated comparison of ortholog assertions made by PhIGs, HomoloGene, Ensembl Compara, Inparanoid and MGI [17,18]. Currently, the HCOP website also includes predictions from Evola, HGNC, OMA, OPTIC, TreeFam, UCSC and ZFIN and has removed PhIGs http://www.genenames.org/cgi-bin/hcop.pl. Another tool, COMPARE, is not limited to human-centered relationships and combines results from Inparanoid, orthoMCL, Ensembl Compara and TreeFam [19]. These combined approaches are useful in that they allow for identification and comparison of ortholog results from various sources, allowing the end-user to make informed choices. However, neither tool incorporates the full range of approaches available (*i.e*. sequence-based, tree-based and network-based approaches) and facilitates comparison among any pair of common model organisms. For example, HCOP only provides predictions relevant to human genes, and COMPARE provides predictions among human, mouse, worm, fly, chicken, zebrafish and sea squirt genes but not yeast genes. Thus, room for significant refinement of integrative approaches remains.

After successful ortholog prediction, a next challenge can be to combine functional information obtained in multiple organisms to uncover conserved functions that provide insights into human diseases. Insight into disease using ortholog prediction tools can be twofold. First, identification of orthologs can make model organisms available to perform molecular genetic studies of conserved disease genes that are not possible to perform in other organisms. Second, conserved genes first identified in a model organism can become candidate disease genes and/or provide new insights relevant to disease. We also note that genes that are not conserved among specific groups of species or have distinct differences may be important for evolutionary studies, and may be good targets for the development of biocides, such as to control disease vector, parasite, or crop pest populations [20,21].

The increasing number of human genes that have been directly or indirectly associated with diseases provides a good starting point for identification of conserved orthologs in model organisms for disease-focused studies. To date, a large number of disease-associated genes have been reported and the information compiled into various resources. Perhaps top among these is the NCBI Online Mendelian Inheritance in Man (OMIM) database [1-4]. Since the completion of the human genome and following various technological advances, an increasing number of genome-wide association studies (GWAS) provide evidence for possible disease association of many previously uncharacterized genes [5,6]. In addition, there have been several automated efforts to evaluate the links between human diseases and genes, such as based on gene annotations or the published literature. The Disease Ontology project, for example, assigns disease information based on GeneRIF annotation [22], and resources such as BITOLA, MedGene and HuGE Navigator [23-25] provide systematic summaries of disease-gene relationships culled from the literature in NCBI PubMed.

Facilitating the identification of orthologs between a model organism and humans is of particular relevance to genes associated with human diseases. *Drosophila *is a model organism of particular interest for which a wide variety of molecular genetic tools are readily available. Moreover, *Drosophila *models have been developed for a number of human diseases. For example, *Drosophila *is an established model for a wide variety of nervous system-related diseases (reviewed in JEIBMANN and PAULUS 2009)[26]; various muscular dystrophies [27-29]; responses to infection by human pathogens (*e.g*. [30-34]); multi-symptom inherited disorders [35,36]; heart disease [25,37,38]; and cancer [9,39-42]. *Drosophila *is an emerging model for the study of asthma [43], lipotoxicity [44], and metabolism [45-47], and is being used to study responses to therapeutic treatments and natural products [48-50]. Overall, conservation between humans and *Drosophila *at the gene, pathway, cognate organ and behavioral levels, together with available tools and assays, suggest that *Drosophila *can serve as an excellent system for rapid functional characterization of GWAS candidates and other disease-associated genes, such as by placing them in specific conserved pathways or implicating them in specific metabolic, physiological or developmental roles.

Despite the utility and potential of *Drosophila *in studying disease, however, the number and scope of resources linking human disease genes to known or putative *Drosophila *orthologs is still limited. OrthoDisease and Homophila are two tools aimed at translation of OMIM disease gene annotations to genes of other organisms. OrthoDisease maps orthologous gene relationships among human genes and genes in 100 genomes, including *Drosophila*, using the Inparanoid tool [51]. Homophila was designed specifically for *Drosophila *and uses BLASTP to compare human protein sequences to the *Drosophila *proteome [52,53]. Both approaches have value and are used extensively. Nevertheless, each has important caveats. For example, OrthoDisease may miss relevant orthologs due to the limited coverage provided by use of just one approach to ortholog mapping, and Homophila can generate inappropriate gene mapping via uncurated one-way BLASTP results. Moreover, neither tool currently accommodates the increasing amount of GWAS data available to the community. Thus, we have developed a new online tool that integrates the results of several ortholog-mapping tools based on different algorithms, and used the approach to create an online-searchable analysis of relationships among genes in OMIM or GWAS data sets and genes in the mouse, zebrafish, *C. elegans, Drosophila*, and *S. cerevisiae *genomes.

## Results and Discussion

### An integrative tool to identify putative orthologs among several model organisms

More than three dozen large-scale ortholog prediction tools or resources have been developed in the past decade, and more than a dozen of these are currently available online in relatively accessible formats. Based on published reviews and comparative studies evaluating different tools, we selected nine tools that provide a reasonable balance of specificity and sensitivity [7-12,15,16,54-59]. We excluded tools that were developed prior to the availability of full-genome sequence and/or that have not been maintained and updated. However, we note that the data available from tools we included are based on different genome releases (see the Documentation Page, http://www.flyrnai.org/DRSC-OPT.html). Three of the nine tools we selected are representative of the phylogenetic tree approach and five are representative of tools that cluster genes based on genome-wide sequence comparison. In addition, the recent availability of large datasets and networks of protein-protein interactions (PPIs) has opened the doors to proteomics-based approaches to identification of functional orthologs, *i.e*. predictions based on the similarity of the PPI networks surrounding the potentially orthologous proteins. A leading resource for ortholog prediction based on this approach is IsoBase [15,60,61], which is aimed at identification of functionally related proteins based on integration of sequence similarity and protein-protein network-based information. As our principle goal in developing the tool is a focus on functional orthologs, we included IsoBase in addition to the other eight tools in our integrative approach.

Integration of the results of these nine tools required a number of pre-processing steps and decisions, as the outputs from the different tools differ both in terms of their use of gene and protein identifiers, and in terms of the format and complexity of output files. The major identifiers used by the nine tools are Ensembl protein identifiers, NCBI gene or protein identifiers, Uniprot identifiers, and species-specific identifiers such as from FlyBase and WormBase (Additional file 1). Another difference among the tools that we had to address was that some tools make predictions based on comparison with all protein isoforms of a gene, whereas others first normalized the proteins at the gene level by selecting the longest isoform for inclusion in the analysis. To facilitate merging and comparing the outputs from the nine tools we included, we first programmed an algorithm to standardize different identifiers to Entrez gene IDs using mapping files, such as from NCBI or species-specific databases. Our use of gene IDs eliminated redundancy due to inclusion by some tools of multiple isoforms. Recognizing that many users of our tool would prefer to be inclusive rather than to inadvertently exclude genes that might be relevant to a given disease-focused or other study, we chose to include not only ortholog predictions but also inparalog or co-ortholog predictions. Although paralogous genes may have diverged in function more than orthologs, those that have arisen from recent gene duplications may be members of a function-oriented ortholog group [57,62]. When building the tool, we first obtained the data from each site; then standardized various identifiers and converted the different output formats; next integrated all the predictions into our FlyRNAi database [63]; and finally launched an online application (see Materials and Methods).

**Figure 1:**
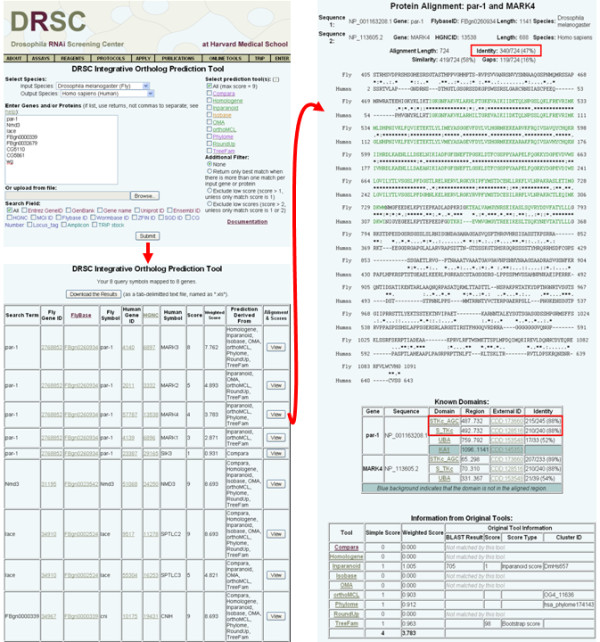
**DIOPT facilitates identification of predicted orthologs based on various approaches, with flexible inputs and outputs**. The DIOPT graphical user interface facilitates input of small or large gene or protein lists and is compatible with one or more of a variety of identifiers. Three filters are available: two of them exclude relationships that are predicted by < 2 or < 3 tools unless the only match score is equal to or lower than the threshold. The third one and most stringent filter limits outputs to the best-matching ortholog(s) per gene, as judged by the number of tools supporting the prediction. DIOPT provides a score based on the number of tools supporting the prediction and a weighted score based on the average of GO semantic similarity of orthologous pairs predicted by each tool using high quality GO functional annotation. In addition, DIOPT also provides the option to view the original score of each tool and a protein-protein alignment based on an updated proteome annotation (RefSeq release 44). Percent protein identity is calculated for both the overall alignment and domain regions.

The new tool is named DIOPT, for DRSC Integrative Ortholog Prediction Tool, and it is freely accessible online at http://www.flyrnai.org/diopt (Figure 1). Using *Drosophila *or human data as inputs, we find that DIOPT identified 28605 fly and human orthologous relationships, of which 2537 are one-to-one relationships. As expected, DIOPT identified more human-fly many-to-one relationships than one-to-many relationships (*i.e*. 5504 versus 1672), consistent with the lower redundancy of the fly genome. DIOPT achieves an approximately 70% to 428% increase in the number of *Drosophila*-human gene relationships identified as compared with use of any individual tool. This corresponds to 20% to 116% more coverage of the *Drosophila *genome and 30% to 172% more coverage of the human genome (Table 1). DIOPT allows users to use all nine tools or any subset in a search. Although a small number of differences in predictions might be due to the use of different genome release versions by different tools, most differences will be due to the specific algorithms and approaches used in the various tools. DIOPT calculates a simple score indicating the number of tools that support a given orthologous gene-pair relationship, such that the maximum score for most species pairs is nine (see the Document Page, http://www.flyrnai.org/DRSC-OPT.html), assuming that the user selects to accept outputs from all tools (*i.e*. the default setting). Because all nine sources cover the entire genome of each species included in the search, we reasoned that this simple count of how many tools agree with each other is an appropriate first indicator of confidence (see below). We next focused on methods for evaluation and improved display of the results, towards the goals of generating higher-confidence outputs, making the tool user-friendly for biologists, and providing additional gene- or protein-associated information and functionality.

**Table 1:** The number of *Drosophila*-human ortholog pairs predicted using individual published ortholog tools or the integrative tool DIOPT

	*D.mel-H.sap* relationships	Unique relationships*	*D.mel *genes covered	*H.sap *genes covered
Compara	16880	3994	8416	9826
Homologene	5423	82	5015	4762
Inparanoid	10204	695	5855	7441
IsoBase	9051	1639	8065	7251
OMA	5673	296	4499	5222
orthoMCL	12853	2352	6435	7890
Phylome	8690	1496	4560	6251
RoundUp	7480	398	5383	6706
TreeFam	12894	1347	7156	9106
DIOPT	28605	NA	9724	12971

D.mel, Drosophila melanogaster; H.sap. Homo sapiens

* Unique Relationships, relationships not predicted by any of the other eight tools.

### Assessment of functional consistency of ortholog pairs predicted using DIOPT

**Figure 2:**
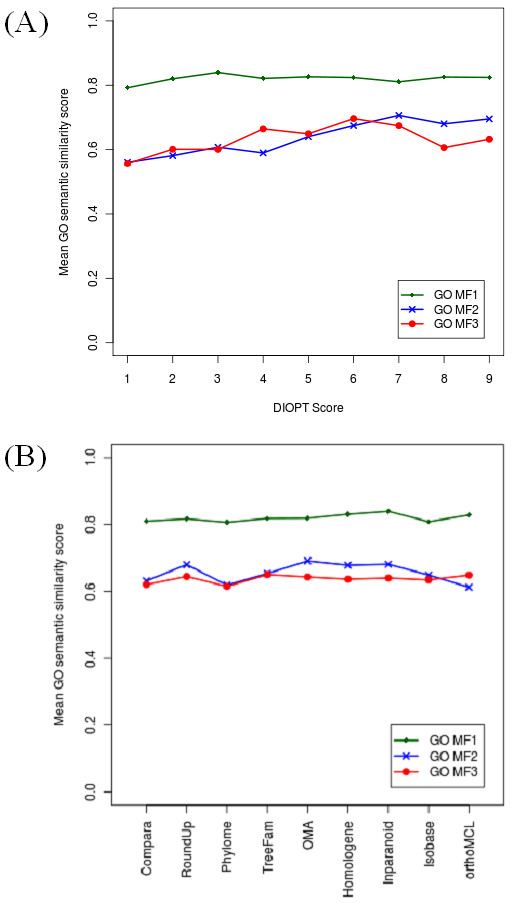
**Assessing the correlation of functional relatedness and DIOPT score with gene ontology molecular function (GO MF) annotations**. GO MF1, all GO annotations of molecular function. GO MF2, the subset of GO annotations of molecular function supported either by experimental data or author/curator statement. GO MF3, the subset of GO annotation of molecular function supported by experimental data. 2a. Correlation of functional relatedness and DIOPT score with GO MF annotations. 2b. Comparison of the functional relatedness of orthologous genes predicted by individual tools.

As the field lacks a commonly accepted "gold-standard" test set for assessment of quality, we opted to test a few approaches that together help give an overall picture of the quality of DIOPT results. Our first assessment was based on molecular function annotation of gene ontology (GO MF). GO MF is widely used to assess the functional consistency of two genes. As a first test of DIOPT performance, we examined the similarity of GO MF annotations for all *Drosophila*-human ortholog pairs predicted using the default DIOPT settings (*i.e*. incorporating results from all nine tools). We recognized that a potential caveat to using GO MF annotations to analyze performance is the danger for 'circular arguments', as some GO MF annotations are themselves based on information about orthologs. Indeed, 40% of GO functional annotations for *Drosophila *genes and 55% for human genes were assigned based on sequence similarity without manual inspection. We were able to address this by taking into consideration the evidence codes for GO MF assignments. Namely, we opted to perform the analysis with two subsets; *i.e*., the subset of GO MF annotations supported by experimental data or curator/author statements, and applying an even more stringent rule, only those GO MF annotations that are supported by experimental data. We used GOSemSim [64], an R package http://www.r-project.org/ for semantic similarity computation, to calculate the functional similarity score for each orthologous pair by considering the set of selected GO terms assigned to each gene. The results of our analysis demonstrate that ortholog pairs with lower scores (*i.e*. predicted by fewer tools) have nearly comparable functional consistency as compared with ortholog pairs with high scores. However, higher score pairs do show a slightly higher level of functional similarity (Figure 2a). Thus, DIOPT scores provide one means of prioritizing or limiting gene list outputs.

The lack of a standardized benchmark remains a significant challenge in comparing and evaluating ortholog prediction tools. The "Quest for Orthologs" initiative is currently attempting to provide benchmarking for orthology predictions but that resource is not yet available [65]. To obtain some measure of DIOPT performance relative to individual tools, we used two different approaches based on available resources. First, we used a manually assembled set of high-confidence *Drosophila*-human orthologous pairs made up of 159 orthologous pairs of trans-membrane proteins generated by manual phylogenetic analysis [54], 89 pairs of kinases generated manually based on kinase domain similarity as well as phylogeny [66] and 71 pairs of oxidative phosphorylation genes assembled manually by sequence alignment and analysis of exon/intron structures [67,68]. All these data sets, with the exception of the OXPHOS set, have been previously used for evaluation of ortholog prediction tools. The idea here was to identify a set of proteins where orthology relationships can be established with high confidence by approaches other than those discussed here. To this end, the dataset's composition is biased towards types of proteins for which such independent verification is feasible. However, such a bias can distort the evaluation results: the coverage of the selected proteins (especially, trans-membrane proteins) in PPI data is low and consequently, the performance of a PPI-based method like IsoBase is likely to be underestimated. Nevertheless, we believe that testing with this set can provide one useful way to ask if as predicted, the integrative approach provides improved results as compared with use of individual tools.

**Figure 3:**
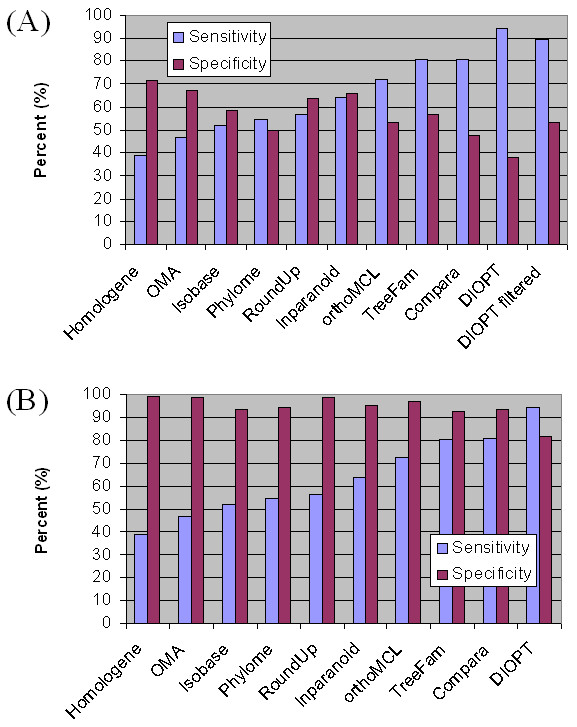
**Comparing sensitivity and specificity among the nine individual ortholog prediction tools and the integrative tool DIOPT**. Comparing sensitivity and specificity using a manually assembled reference set (3a), or using the KOG human-specific gene set (3b). Sensitivity is defined as the percent of manually assembled pairs that can be identified by each tool versus all the manually assembled orthologous pairs (same for Figures 3a and 3b). In 3a, specificity is defined as the percent of manually assembled pairs that can be identified by each tool versus all the orthologous pairs identified by each tool if queried with either the *Drosophila *or the human genes from the test set. In 3b, specificity is defined as the percent of putative human-specific genes that do not have fly ortholog versus all the human specific genes from KOG list.

DIOPT is able to identify 301 of the total of 319 relationships in the high-confidence manually assembled test set. Thus, DIOPT achieved 94% sensitivity, comparing favorably to the 39% to 81% sensitivity observed with individual tools. We also used this test set to look at specificity and observed the classical trade-off between specificity and sensitivity. (See Figure 3 for definitions of these terms.) Without filters, the specificity of DIOPT is 38%, as compared to 48% to 71% observed with individual tools (Figure 3a). This decrease may be acceptable for some applications of the tool; however, for other applications, users may prefer to limit the results. As mentioned above, we reasoned that the simple DIOPT score (*i.e*. the number of tools supporting the prediction) might provide a way to achieve higher specificity, and this indeed proves true. If we filter out the relationships with score equal to one, *i.e*. relationships supported by only one of the 9 tools, the sensitivity of DIOPT remains at about 90% but specificity increases from 38% to 53%. To take best advantage of this or of a user's own assessment, we provide users with several options for filtering results. Users can include any subset of the nine tools in a search; apply a filter that removes predictions supported by only one tool (*i.e*. scores < 2 are removed); or apply a filter that returns only the predictions with the best score (*i.e*. if a query gene matches to more than one ortholog, only the ortholog(s) with the highest score are displayed).

We performed a different type of test for specificity using a negative test set; *i.e*. a set of 1,130 human genes defined as human lineage-specific by Eukaryotic Clusters of Orthologous Groups (KOG) [37]. We found that DIOPT shows lower specificity (82%) as compared to individual tools using this test set (93%-99%) (Figure 3b). This may be partially due to the fact that the 'lineage-specific' set in KOG was assembled over a decade ago, when the available approaches may not have been sensitive enough to detect meaningful gene relationships that exist in this group. This idea is supported by the observation that 34% of the genes from the KOG set that were identified as not human specific by DIOPT are predicted by multiple tools to have orthologs in the fly.

### Online Features of DIOPT

More than a dozen ortholog prediction tools are currently available online. However, most tools do not allow for searching in batch mode (*i.e*. with a long list of genes or other identifiers), making them impractical to use for many types of analysis, including analysis of the typically long lists of genes or proteins resulting from the increasing number of high-throughput RNAi, proteomics and other studies. Although many of the tools do allow a researcher to install the program locally, potentially facilitating batch searches, this is a significant hurdle for a researcher without expertise in bioinformatics, and even after installation, large datasets may still be difficult to process and subsequently manage as outputs. To make searching with short or long lists of identifiers possible online, we developed DIOPT to have the flexibility of accepting one or a few typed entries, a long list of entries, or an uploaded list of gene or protein identifiers (Figure 1). Moreover, we built gene and protein mapping into DIOPT and accommodate a large number of NCBI, species-specific and other different identifiers. Thus, after choosing the input and output species of interest, researchers can use most common identifiers, or even mix different types of identifiers, in their input lists. For expert users, we also provide the option of specifying the type of identifier, such that other matches may not be included. We also make it possible to search with RNAi reagent IDs (i.e. amplicon IDs) from the *Drosophila *RNAi Screening Center (DRSC) or fly stock IDs from the Transgenic RNAi project (TRiP; http://www.flyrnai.org/TRiP-HOME.html) [69,70].

With DIOPT, users can search with many ID types and either includes output from all tools with any score or limit outputs (see above and Figure 1). The score > 1 and score > 2 filters are currently configured to provide a low score match if it is the only match to an input. These results can easily be excluded, such as by sorting data by that column following download. To further help users evaluate orthologs we have also calculated weighted scores. To do this, the nine tools were weighted based on average GO semantic similarity of orthologous pairs annotated with high-quality GO MF annotation (GO MF2, see above). The average GO semantic similarity scores are very similar for each of the 9 tools, ranging from 0.612 to 0.691 (Figure 2b). To assign weights, we normalized the scores by setting the median to one and defined the weights accordingly (0.903 to 1.019). Weighted scores serve as an additional way for users to make informed decisions when multiple orthologs with the same simple count score are retrieved.

We have added the option to view the original prediction/evaluation scores from each of the tools, along with the protein alignment. To help address the caveat that the original predictions were made by various tools using different genome releases, the protein-protein alignments we provide are based on a current proteome annotation for any given output pair, including highlighting of regions that correspond to domain annotations (RefSeq release 44; Figure 1). The sequence identities of the entire aligned sequence, as well as identity within the annotated domain regions, which typically have higher identity, are summarized and displayed. For example, for the fly gene *par-1 *(FlyBase ID FBgn0260934) and its human ortholog MARK4 (NCBI Gene ID 57787), DIOPT shows that the overall protein identity is 47% whereas the identity of the kinase catalytic domain is 88% (Figure 1). This feature allows researchers to quickly zoom in on functionally critical motifs, amino acids, *etc*. based on their specific expertise and interests. Finally, the database and application of DIOPT were designed in a way that it is easy to be updated and expanded, such as when new and improved ortholog prediction tools become available, or when existing tools are updated.

### Using DIOPT results to identify genes in model organisms related to human diseases

High-throughput genomics technologies generate a vast amount of data, making it possible for researchers to search for genetic causes of human diseases. Concomitant with the rise in disease genomics is an increase of publicly accessible resources that associate loci, mutations and genes with putative or known roles in disease. Perhaps top among these is Online Mendelian Inheritance in Man (OMIM), which focuses on inherited diseases. The information contained in OMIM has been extracted from publications and undergone manual curation [1-4]. GWAS data sets, such as those cataloged by the National Genome Research Institute (NHGRI; see http://www.genome.gov/gwastudies/), provide associations among loci or genes and diseases or traits based on linkage analysis using single nucleotide polymorphisms as markers. Due to technological advances in genotyping, the amount of GWAS data now available has increased dramatically [5,6]. With GWAS data, identification of a specific disease-causing mutation in a gene may be missing, with the results pointing to one or more candidate genes that may be relevant. These candidates require further functional characterization, and the wealth of phenotypic information available in *Drosophila *and other model organisms may be of help to narrow down the list of genes in a disease-associated locus.

*Drosophila *has been used extensively to study human diseases but as discussed previously, resources linking fly or other model organism genes to human diseases are still limited. The tools OrthoDisease and Homophila were developed to help identify disease genes in model organisms. These tools incorporate OMIM but not GWAS information and their use of OMIM data sets differ from one another. OMIM annotation includes both gene/locus terms and disease phenotype terms. OrthoDisease includes most phenotype terms, whereas Homophila is focused on gene/locus terms, with neither tool fully incorporating all available terms at OMIM. Moreover, OrthoDisease uses Inparanoid predictions to map human genes to genes in model organisms, such that some ortholog pairs identified using DIOPT-DIST are missed when searching OrthoDisease (*e.g*. mapping of human TP53 to *Drosophila *p53). By contrast, Homophila uses one-way BLASTP, which is so inclusive that it tends to include erroneous predictions. For example, ALDH2 is identified in OMIM as 'causing acute alcohol sensitivity' (OMIM IDs 610251 and 100650). For ALDH2, OrthoDisease predicts one corresponding gene in *Drosophila *(*Aldh*) and Homophila predicts 11 corresponding genes in the fly. In contrast, DIOPT predicts two fly genes: *Aldh*, which is also predicted using OrthoDisease and Homophila, and the additional gene *CG31075*, which shares 59% identity and 74% similarity with human ALDH2 and is predicted to be an ALDH2 ortholog by four tools.

**Figure 4:**
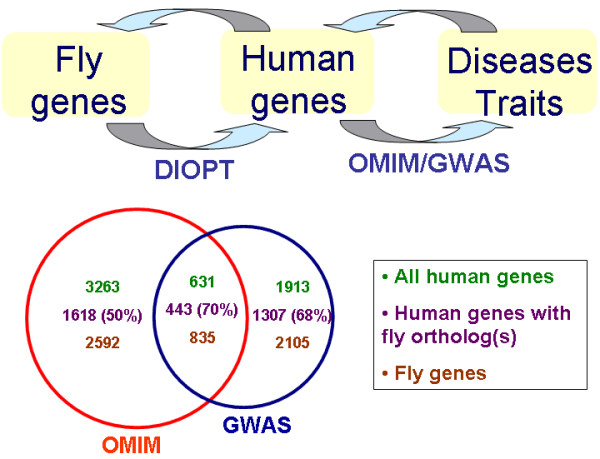
**Identification of predicted disease gene orthologs in model organisms**. 4a. Strategy for identifying disease genes in model organisms. 4b. Summary of disease genes from OMIM and GWAS and predicted orthologous relationships for *Drosophila*.

**Figure 5:**
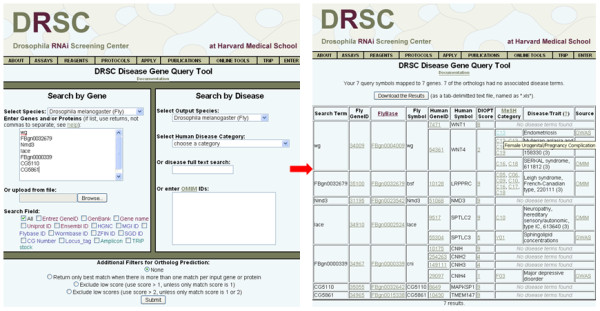
**User Interface for the disease and trait query tool DIOPT-DIST**. Users can query with a list of genes from model organisms, and a list of human orthologs along with their disease annotations from OMIM/GWAS will be retrieved. Alternatively, users can query with a disease term, disease category or OMIM IDs (OMIM IDs for disease phenotype and/or gene/locus), and a list of human genes as well as their corresponding orthologs in a model organism will be retrieved. The DIOPT score is displayed on the results page and is hyper-linked to detailed information including a protein alignment, the original tool scores, and the weighted DIOPT score.

Thus, we reasoned that developing a tool based on DIOPT might improve upon results from existing tools, as well as provide an opportunity to incorporate newly available GWAS data. The tool we developed was named DIOPT-DIST as it combines DIOPT ortholog mapping with disease and trait information http://www.flyrnai.org/diopt-dist. With DIOPT-DIST, we linked *C. elegans*, *Drosophila*, zebrafish, mouse or yeast genes to human diseases by extracting disease-gene information at OMIM http://www.ncbi.nlm.nih.gov/omim and GWAS data cataloged by NHGRI http://www.genome.gov/gwastudies and then using DIOPT to map orthologs (Figure 4 &5; see also Materials & Methods and the Documentation Page, http://www.flyrnai.org/DRSC-OPT.html). DIOPT-DIST includes both disease phenotype terms and gene/locus terms from OMIM. At the DIOPT-DIST website, users can query with one or more genes from a model organism to get a list of human orthologs along with their disease or trait annotations. Conversely, users can query with a disease term, disease category (see below) or OMIM IDs to get a list of human genes as well as their corresponding orthologs in a specified model organism. The DIOPT score in the results table links to a page displaying a protein alignment and details about the DIOPT results.

To develop the DIOPT-DIST tool, we first had to normalize the disease and trait information using a standardized classification of disease terms. The 5700 disease or trait descriptions from the OMIM and GWAS data sets are in free text and show significant variation in word choice, phrasing and sentence structure in describing diseases. Indeed, in some cases the terminologies vary even within one resource, including spelling variations or typing errors. These variations hinder systematic searches for disease phenotypes. By contrast, Medical Subject Heading (MeSH) terms are a previously established controlled vocabulary describing disease phenotypes (C01-C27) and mental disorders (F03) with a hierarchal structure allowing more general disease terms to be distinguished from sub-classes. MeSH accommodates most disease synonyms and is used to index medical literature in PubMed [71]. Thus, we chose to classify OMIM and GWAS disease terms based on MeSH general disease categories in order to facilitate disease search and data mining.

To do this, we took advantage of OMIM clinical synopsis heading/sub-heading text [72], and were able to map about 3000 OMIM disease terms to the corresponding MeSH terms. Using MeSH synonym annotation, we were able to classify most of the remaining OMIM and GWAS terms. Some cannot be mapped using MeSH clinical synopsis heading/sub-headings or synonyms because they are traits rather than diseases (*e.g*. eye or hair color) or they are disease risk factors, diagnoses or treatments, and neither of those categories is covered by MeSH disease annotations. Therefore, we created two additional categories, "Disease risk factor, diagnosis or treatment" and "Trait", and mapped to these categories as appropriate (Table 2). Because both OMIM and GWAS are updated periodically, and particularly as GWAS data is accumulating at a rapid pace, we plan to regularly update DIOPT-DIST, including conversion to our standard MeSH-based terms. The date of the last update will be indicated on the DIOPT-DIST search page.

**Table 2:** Disease genes from OMIM and GWAS (sorted by # of human genes)

Disease category	# of disease terms	# of human genes	# of *Drosophila *genes	# of *Drosophila *genes filter1	# of *Drosophila *genes filter2
Congenital, Hereditary, and Neonatal Diseases and Abnormalities	3518	2803	2614	2076	1890
Nervous System Diseases	1921	1816	1901	1437	1339
Disease risk factor, diagnosis or treatment	273	1459	1768	1398	1292
Musculoskeletal Diseases	1523	1332	1433	1110	1035
Eye Diseases	1410	1214	1356	1038	953
Skin and Connective Tissue Diseases	1100	1117	1349	1039	949
Digestive System Diseases	901	1063	1397	1058	935
Otorhinolaryngologic Diseases	933	879	996	750	704
Male Urogenital Diseases	782	863	1069	838	765
Cardiovascular Diseases	843	856	1140	869	756
Female Urogenital Diseases and Pregnancy Complications	771	830	1041	819	741
Nutritional and Metabolic Diseases	490	701	896	698	608
Stomatognathic Diseases	686	637	761	559	528
Mental Disorders	344	626	906	610	543
Neoplasms	500	616	673	493	463
Pathological Conditions, Signs and Symptoms	539	608	489	337	319
Hemic and Lymphatic Diseases	628	584	775	614	545
Endocrine System Diseases	442	511	627	451	427
Immune System Diseases	323	480	523	371	337
Respiratory Tract Diseases	447	479	639	493	445
Trait	208	434	536	374	336
Virus Diseases	23	65	48	41	39
Bacterial Infections and Mycoses	19	32	36	24	24
Parasitic Diseases	7	16	28	19	18
Substance-Related Disorders	7	14	17	13	13

We found a total of about 6000 human genes from OMIM and GWAS data associated genetically with a wide range of diseases and traits. Using DIOPT without any filter, we can identify 4282 corresponding orthologs in *Drosophila*. The numbers drop to 3620 and 3382 *Drosophila *genes, respectively, when we apply filters that exclude predictions supported by scores of < 2 or < 3. Of these high-confidence orthologs, 33% were from both OMIM and GWAS, 37% were from OMIM only, and 30% were from GWAS only (Figure 4). Our results suggest that molecular functions relevant to a large number of human disorders and GWAS associations could potentially be studied using *Drosophila*. Table 2 summarizes the distribution of these *Drosophila *genes over 25 disease categories in MeSH, plus our two added categories. The categories with the most genes are "Congenital, Hereditary, and Neonatal Diseases and Abnormalities", "Nervous System Diseases", "Disease risk factor, diagnosis or treatment" and "Musculoskeletal Diseases". As expected, DIOPT-DIST also performs well with examples such as those mentioned above, *i.e*. human TP53 mapping to *Drosophila *p53 (*e.g*. p53 appears among results of a disease full text search for "cancer" with *Drosophila *as the output species) and the mapping of ALDH2 to *Drosophila Aldh *and *CG31075 *(*e.g*. resulting from search with OMIM ID 610251).

The annotated results of our analysis should be a useful resource for prioritizing genes from *Drosophila *and four additional model organisms (*S. cerevisiae, C. elegans*, zebrafish, or mouse). Users can query with a disease or disease category to generate a corresponding list of predicted orthologs of the human genes in one of the five model organisms included in the tool (Figure 5). For example, a search using "diabetes" currently identifies 217 predicted Drosophila orthologs corresponding to human diabetes-related genes (see Additional file 2). This list includes 30 genes that have previously been studies in metabolic diseases in fly, including *Akt1*, *InR *and *chico *(reviewed in [45,73]). This list also provides additional candidates such as *sima *and *Spargel*. The availability of a wealth of molecular genetic tools for *Drosophila *makes it possible to study candidate disease-associated genes identified using DIOPT-DIST.

### Comparing disease associations in the literature for Drosophila and human orthologs

In addition to curated sources like OMIM, information about gene and disease associations also exist in the form of millions of published literature citations in free text format. For example, Bodenreider and Burgun recently showed that the MeSH index of biomedical articles provides a framework for comparing disease phenotypes of mouse and human orthologous genes [71]. Moreover, they showed that the categories "Neoplasms" and "Nervous System Diseases" have the most disease annotations in common between mouse and human orthologs. For our analysis, we extracted disease phenotypes based on MeSH indexing of biomedical articles in PubMed literature citations for the 28605 orthologous gene pairs between *Drosophila *and humans (Table 1). We identified 3557 fly genes with at least one disease phenotype associated in the literature. Of these, 2739 (77%) have at least one putative human ortholog as revealed by DIOPT. In addition, 2283 *Drosophila *genes share at least one disease category with their human orthologs based on independent studies (Additional file 3). These *Drosophila *genes cover 23 of the 26 disease categories (Table 3). "Cancer", "Digestive system diseases" and "Nervous system diseases" have the highest percentages of orthologous genes sharing the same disease phenotype(s), whereas parasitic diseases, eye diseases and infectious diseases have the lowest percentages of orthologous genes with the same disease phenotype(s).

**Table 3:** Disease phenotypes associated with *Drosophila *genes in the literature

Mesh	# of fly genes	# of fly genes with human ortholog(s)	# of fly genes shared disease categories with their human ortholog	% fly genes with human ortholog(s)	% fly genes shared disease categories with their human ortholog
Neoplasms	681	636	592	93%	93%
Pathological Conditions, Signs and Symptoms	2105	1713	1566	81%	91%
Nervous System Diseases	1179	1004	878	85%	87%
Digestive System Diseases	105	94	81	90%	86%
Otorhinolaryngologic Diseases	18	14	12	78%	86%
Mental Disorders	470	448	378	95%	84%
Congenital, Hereditary, and Neonatal Diseases and Abnormalities	1234	1100	900	89%	82%
Cardiovascular Diseases	139	126	103	91%	82%
Stomatognathic Diseases	20	20	16	100%	80%
Skin and Connective Tissue Diseases	148	123	98	83%	80%
Immune System Diseases	112	84	64	75%	76%
Respiratory Tract Diseases	8	8	6	100%	75%
Disorders of Environmental Origin	760	642	466	84%	73%
Hemic and Lymphatic Diseases	100	96	69	96%	72%
Endocrine System Diseases	163	144	103	88%	72%
Nutritional and Metabolic Diseases	577	452	310	78%	69%
Female Urogenital Diseases and Pregnancy Complications	558	413	276	74%	67%
Virus Diseases	38	32	21	84%	66%
Musculoskeletal Diseases	211	165	106	78%	64%
Male Urogenital Diseases	468	355	221	76%	62%
Bacterial Infections and Mycoses	325	233	99	72%	42%
Eye Diseases	699	590	221	84%	37%
Parasitic Diseases	229	187	21	82%	11%
All Categories	3557	2739	2283	77%	83%

We further looked at the publications for the 4282 fly disease genes identified by mapping human OMIM and GWAS data. 1534 of them have been published as related to disease using the fly model and 888 (58%) are associated with the same disease category in the literature as that assigned by OMIM/GWAS mapping (Table 4). This supports the idea that orthologous genes are likely to have similar disease phenotypes across species and also validates disease category assignments made when we mapped OMIM/GWAS annotation. This analysis also demonstrates that there are still many disease candidate genes to be studied in model organisms. OMIM and GWAS annotation are ongoing efforts, enriching for genes mutated in the germ-line and associated with inherited human diseases, traits, predisposition, *etc*. Conversely, literature mining uses existing information to identify additional disease-gene associations, and provided more than 2000 additional candidate disease-related genes in *Drosophila*, a useful supplement to the set of genes annotated at OMIM and GWAS.

**Table 4:** Comparison of *Drosophila *disease genes identified from OMIM/GWAS and fly disease genes identified by literature mining

Disease Category	Fly disease genes mapped from OMIM/GWAS	Fly disease genes from OMIM/GWAS that are studied in disease in literature	Fly disease genes from OMIM/GWAS with disease categories confirmed by literature	% of fly disease genes with disease categories confirmed by literature
Congenital, Hereditary, and Neonatal Diseases and Abnormalities	2614	707	539	76%
Otorhinolaryngologic Diseases	996	12	8	67%
Nervous System Diseases	1901	669	431	64%
Musculoskeletal Diseases	1433	88	56	64%
Cardiovascular Diseases	1140	95	58	61%
Stomatognathic Diseases	761	14	8	57%
Skin and Connective Tissue Diseases	1349	72	36	50%
Digestive System Diseases	1397	66	31	47%
Hemic and Lymphatic Diseases	775	64	27	42%
Neoplasms	673	382	145	38%
Male Urogenital Diseases	1069	202	75	37%
Eye Diseases	1356	334	120	36%
Respiratory Tract Diseases	639	6	2	33%
Immune System Diseases	523	56	18	32%
Female Urogenital Diseases and Pregnancy Complications	1041	236	72	31%
Mental Disorders	906	345	105	30%
Endocrine System Diseases	627	94	27	29%
Nutritional and Metabolic Diseases	896	255	71	28%
Parasitic Diseases	28	122	2	2%
Pathological Conditions, Signs and Symptoms	489	1074	143	13%
Bacterial Infections and Mycoses	36	133	14	11%
Disorders of Environmental Origin	1768	358		0%
Virus Diseases	48	24	0	0%
Substance-Related Disorders	17		0	0%
All Categories	4282	1534	888	58%

## Conclusions

Existing approaches to identification of predicted orthologs provide an excellent starting point for the development of integrative tools. DIOPT provides a simple but effective method for integrating ortholog predictions and viewing the results in user-friendly formats. DIOPT-DIST builds on this further by filling a growing need to build links from human diseases to genes to putative orthologs in model organisms such as *Drosophila *that can be used to analyze gene function. Various sources of test sets provide ways to assess the quality of the tools and demonstrate the utility of both DIOPT and DIOPT-DIST. These tools and approaches should be particularly useful to researchers interested to use model organisms to study known or putative human disease genes.

## Methods

### Data source

Genome-wide orthologous prediction results of the 9 tools selected were retrieved from their corresponding websites (see the Document Page, http://www.flyrnai.org/DRSC-OPT.html). The test set of manually assembled orthologous pairs was retrieved from three resources and fly-human relationships were selected. The trans-membrane proteins were retrieved from Multi-Paranoid http://multiparanoid.sbc.su.se/stats.html; protein kinase annotations were retrieved from OrthoInspector http://www.ncbi.nlm.nih.gov/pmc/articles/PMC3024942/bin/1471-2105-12-11-S1.XLS; and the oxidative phosphorylation nuclear genes were extracted from MitoComp2 http://www.mitocomp.uniba.it/. The set of putative human-specific genes were retrieved from KOG http://www.ncbi.nlm.nih.gov/COG/. Human disease genes were extracted from OMIM morbidmap and mim2gene at NCBI. A clinical synopsis for each disease was extracted from the omim.txt file using an in-house developed JAVA program. GWAS information was extracted from the NGRI GWAS publications list http://www.genome.gov/gwastudies/, which can be downloaded via FTP http://www.genome.gov/admin/gwascatalog.txt[5]. The MeSH entry term lookup file was generated using an in-house developed JAVA program from a MeSH annotation file (d2011.bin), downloaded at the MeSH web site http://www.nlm.nih.gov/mesh/filelist.html. Gene and literature associations were extracted from gene2pubmed at NCBI ftp://ftp.ncbi.nih.gov/gene/DATA/. Disease MeSH indexes were extracted from a local copy of PubMed with an in-house developed JAVA program.

### Categorizing the disease terms

CS headings of OMIM terms were manually matched to MeSH disease category terms. For GWAS disease/trait terms and OMIM phenotype terms without CS headings, terms were first simplified and then mapped to MeSH using the MeSH entry term lookup file (*e.g*. "breast cancer", "breast tumor" are the entry terms for the MeSH term "breast neoplasms"). Terms corresponding to traits (*e.g*. "hair color") or disease risk factors, diagnoses and treatments (*e.g*. terms as divergent as *"*HDL cholesterol," "hip bone size," "telomere length," and "tanning") could not be mapped in this way because they are not covered by MeSH terms (*i.e*. they are not diseases). Therefore, we created two additional MeSH term-like categories, Y01 for "Disease risk factor, diagnosis or treatment" and Y02 for "Trait," and mapped terms to these categories as appropriate (Table 2). Following mapping to existing MeSH categories and the two new categories, about 100 terms remained unmapped. These turned out to be rare disorders and were mapped to MeSH terms manually.

### Implementation of the DIOPT user interface and disease gene query interface

The DIOPT user interface is implemented as a collection of CGI scripts written in Perl. They are hosted on a shared server provided by the Research IT Group (RITG) at Harvard Medical School. The database is hosted on a MySQL server also provided by RITG.

### Protein alignments

Protein sequences were retrieved from the NCBI RefSeq database release 44. The longest isoform was selected if there were multiple isoforms per gene. Protein alignments were computed in advance using the EMBOSS water program and stored in the database for display. The water program was run using the BLOSSUM62 matrix and all defaults. The alignment display features the aligned portion of the two protein sequences with a match line indicating the degree to which each base pair contributes to the alignment score, with "*" indicating an exact match between the bases; ":" indicating a base pair that scores positively on the BLOSSUM62 matrix; "." indicating an alignment between any other two bases, and a space indicating an area where a gap was inserted into one sequence or the other. CDD domain annotations were extracted from RefSeq protein records and are highlighted in green on the aligned sequences. The locations of the domains on the alignment sequences, and the identity scores of the domain regions and the entire alignment, are computed at the time of display.

## List of Abbreviations

DRSC: *Drosophila *RNAi Screening Center; DIOPT: DRSC Integrative Ortholog Prediction Tool; DIOPT-DIST: DIOPT ortholog mapping and Disease and Trait information tool; OMIM: Online Mendelian Inheritance in Man; GWAS: Genome-Wide Association Study; MeSH: Medical Subject Heading.

## Authors' contributions

YH designed and tested the application, implemented the back-end of the application, and drafted the manuscript. IF implemented the user interface and contributed to database design. AV contributed to tool evaluation. CB contributed to disease classification and manuscript editing. BB participated in the design of the application and manuscript editing. NP provided critical input on key features and edited the manuscript. SEM provided oversight, guided development and testing, and helped draft the manuscript. All authors read and approved the final manuscript.

## Supplementary Material

Additional file 1 **summary of different identifiers used by different ortholog prediction tools**. this file shows that the gene and/or protein identifiers used by different tools vary a lot. Click here for file

Additional file 2 **diabetes genes in fly by DIOPT-DIST search**. this file shows the fly orthologs of human genes that associated to diabetes, identified by key word (diabetes) search at DIOPT-DIST. Click here for file

Additional file 3 **the MeSH disease headings shared by fly and human orthologs in literature**. this file contains a list of fly genes that are co-cited with MeSH disease term(s) in PubMed literature and share at least one disease term with their human ortholog(s). Click here for file
