# Supplementary material for: An integrative approach to ortholog prediction for disease-focused and other functional studies
Source: BMC Bioinformatics. 2011 Aug 31;12:357. doi: 10.1186/1471-2105-12-357 (PMC3179972; doi:10.1186/1471-2105-12-357)
Supplement: Additional file 1 — summary of different identifiers used by different ortholog prediction tools. this file shows that the gene and/or protein identifiers used by different tools vary a lot. [file 1471-2105-12-357-S1.DOC]

**Supplementary Table 1.** Gene and protein identifiers used by the nine ortholog prediction tools included in DIOPT.

| Tool | Human | Mouse | | Zebrafish | Fly | Worm | Yeast |
| --- | --- | --- | --- | --- | --- | --- | --- |
| Compara | Ensembl Protein/Gene ID | Ensembl Protein/Gene ID | | Ensembl Protein/Gene ID | Flybase Gene/Protein ID | Locus_tag | Uniprot  Locus_tag |
| Homologene | RefSeq Protein_GI and Accession; Entrez Gene ID | | | | | | |
| InParanoid | Ensembl Protein_ID | | Ensembl Protein_ID | Ensembl Protein_ID | Flybase Protein_ID | WormBase Protein_ID | Locus_tag |
| IsoBase | Internal ID mapped to GenBank/RefSeq Protein Accession; Entrez Gene_ID; Ensembl Gene/Protein ID; Uniprot ID; Flybase Gene_ID; HGNC; MGI; SGD; WormBase | | | | | | |
| OMA | Internal ID mapped to Ensembl Gene/Protein ID; GenBank Protein Accession and Uniprot ID | | | | | | |
| orthoMCL | Ensembl Protein_ID | | Ensembl Protein_ID | Ensembl Protein_ID | Flybase Protein_ID | WormBase Gene_ID | Locus tag |
| Phylome | Uniprot ID  Ensembl Protein_ID | | Uniprot ID  Ensembl Protein_ID | Uniprot ID  Ensembl Protein_ID | Ensembl Protein_ID  FlyBase Protein_ID | Uniprot  Locus_tag | Uniprot  Locus_tag |
| RoundUp | GenBank Protein_GI | | GenBank Protein_GI | Ensembl Protein_ID | GenBank Protein_GI | GenBank Protein_GI | GenBank Protein_GI |
| TreeFam | Ensembl Gene_ID | | Ensembl Gene_ID | Ensembl Gene_ID | Flybase Gene_ID | Locus_tag | Locus_tag |
